# Abdominophrenic Dyssynergia: A Narrative Review

**DOI:** 10.14309/ajg.0000000000002044

**Published:** 2022-09-30

**Authors:** John A. Damianos, Sanjeevani K. Tomar, Fernando Azpiroz, Elizabeth Barba

**Affiliations:** 1 Department of Internal Medicine, Yale New Haven Health System, Yale School of Medicine, New Haven, Connecticut, USA;; 2 Department of Internal Medicine, University at Buffalo, Jacobs School of Medicine and Biomedical Sciences, Erie County Medical Center, David K. Miller Building, Buffalo, New York, USA;; 3 Digestive System Research, University Hospital Vall'Hebron, Centro de Investigación Biomédica en Red de Enfermedades Hepáticas y Digestivas (Ciberehd), Universitat Autónoma de Barcelona, Bellaterra (Cerdanyola del Vallès), Spain;; 4 Gastroenterology and Motility Department, Hospital Clinic of Barcelona, University of Barcelona, Barcelona, Spain.

## Abstract

Chronic bloating and abdominal distension are common and highly bothersome gastrointestinal symptoms. Although the differential diagnoses for bloating and distension are broad, these symptoms are frequently associated with disorders of the gut-brain interaction. Functional abdominal bloating may be a result of visceral hypersensitivity, whereas abdominal distension seems to be a somatic behavioral response associated with abdominophrenic dyssynergia, featuring diaphragmatic contraction and abdominal wall relaxation. We review the available literature regarding abdominophrenic dyssynergia and comment on its epidemiology, diagnosis, treatment, and avenues to address in the near future.


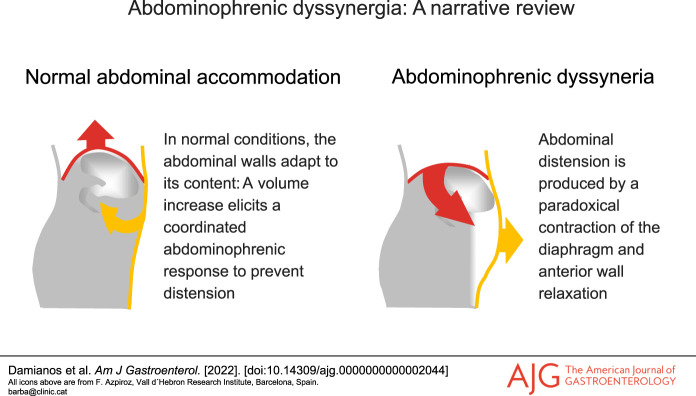


## INTRODUCTION

**Figure 1.**
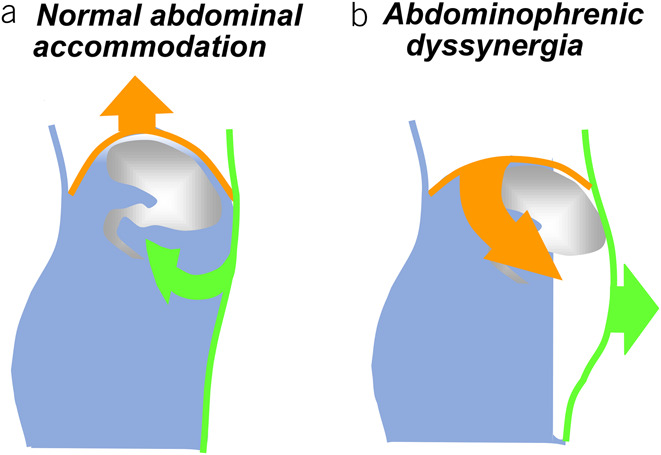
Schematic representation of abdominophrenic interactions. (**a**) In normal conditions, the abdominal walls actively adapt to its content: A volume increase elicits a coordinated relaxation of the diaphragm and contraction of the anterior wall to expand the abdominal cavity without anterior protrusion. (**b**) In patients, abdominal distension is produced by a paradoxical contraction of the diaphragm and anterior wall relaxation.

Chronic bloating and abdominal distension are common in the general population and particularly common in disorders of the gut-brain interaction (DGBIs). Although there are many causes of bloating in DGBIs, a series of studies by Azpiroz et al. indicated that abdominal distension in patients with functional gut disorders is by and large mediated by abdominophrenic dyssynergia (APD) without substantial increments in intra-abdominal contents (1–7). In APD, an abnormal somatic response causes paradoxical movement of the chest and abdominal wall that results in abdominal distension (Figure 1). This response is often triggered by the sensation of bloating. In this review, we summarize the current understanding of APD and what is needed to better serve patients with this disorder.

## BACKGROUND

Bloating refers to the subjective sensation of abdominal fullness, pressure, or gas and has been recently defined as the sensation of increased abdominal pressure/tension, whereas distension describes the objective (visible) increase in abdominal girth. These 2 phenomena are often associated (and frequently conflated), but up to 50% of the time, bloating occurs without distension (8). Occasional bloating and distension are common in the general population, with a prevalence of approximately 40% (9). Although bloating and distension are associated with a wide range of pathologies (both gastrointestinal and nongastrointestinal) (Table 1), these symptoms are particularly common in DGBIs such as irritable bowel syndrome (IBS) and functional dyspepsia (FD). Indeed, up to 90% of patients with IBS and 85% of patients with DGBIs regularly experience these symptoms (8,10). Multiple mechanisms may contribute to bloating and distension in DGBIs, including dysbiosis, small intestine bacterial overgrowth, dysmotility, pelvic floor dysfunction, aerophagia, abnormal gas handling, carbohydrate intolerance or malabsorption, visceral hypersensitivity, central sensitization, and abnormal viscerosomatic responses (11,12).

**Table 1.**
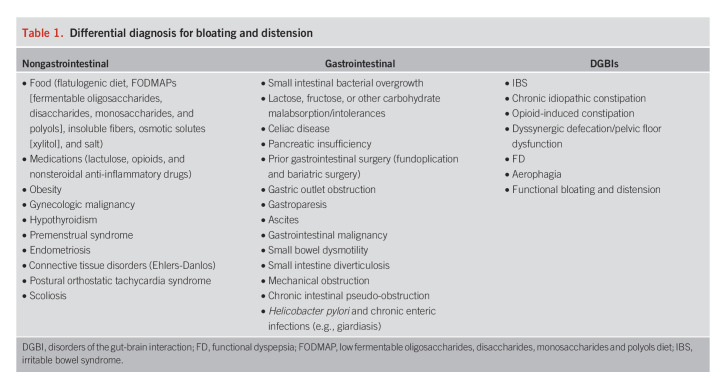
Differential diagnosis for bloating and distension

**Figure 2.**
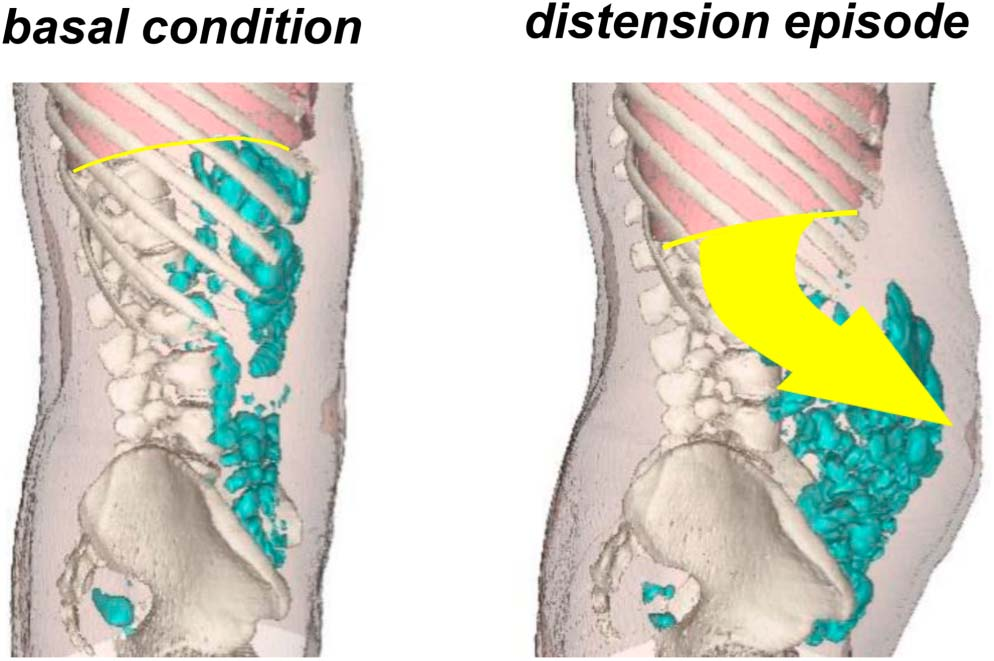
Example of abdominal distension. Three-dimensional reconstructions of CT scans obtained in the same patient during basal conditions and during an episode of abdominal distension. Note diaphragmatic descent and abnormal redistribution of abdominal content associated with distension. CT, computed tomography.

Abnormal viscerosomatic responses seem to be a key contributor to bloating and distension in DGBIs. This phenomenon was first identified by the Barcelona group when a small cohort of patients with either IBS or functional bloating developed abdominal pain and distension associated with dystonia of the abdominal wall when they were challenged with rectal gas infusion (1). Specifically, the abdominal wall failed to contract and there was paradoxical relaxation of the internal oblique muscle. This abnormal response (which was not seen in healthy controls) explained the exaggerated abdominal distension in response to intestinal gas infusion (1). A subsequent study used computed tomography (CT) to compare intestinal gas volumes in healthy controls, in those with DGBIs, and in patients with small bowel dysmotility (chronic intestinal pseudo-obstruction) (2). During reported periods of bloating, patients with dysmotility had increased intestinal gas volumes and anterior protrusion of the abdominal wall, which is consistent with the impaired intestinal motor function. Patients with IBS and FD, however, had no increase in gas volume but did show a diaphragmatic descent and caudoventral redistribution of the abdominal wall and intestinal contents (2) (Figure 2). The role of the diaphragm in bloating and distension was also demonstrated in a cohort of patients with either functional bloating or IBS with constipation (3). Electromyography (EMG) identified that abdominal distension in these patients was driven by paradoxical contraction of the diaphragm and relaxation of the internal oblique muscle, and the term abdominophrenic dyssynergia was coined for this condition (3). The same pathophysiology was subsequently demonstrated in patients with FD complaining of distension: In patients, abdominal distension in response to a test meal was related to APD; by contrast, the test meal in healthy subjects induced a coordinated relaxation of the diaphragm and anterior wall contraction (4). Furthermore, ingestion of lettuce (which is a low gas-releasing substrate for microbial fermentation) led to abdominal distension, produced by uncoordinated activity of the abdominal wall, in patients who believed that eating lettuce would cause them gas and bloating (5), suggesting that distension is a behavioral response. More recently, a study in healthy volunteers demonstrated that voluntary contraction of the diaphragm is associated with abdominal distension and symptoms of bloating and abdominal discomfort (13).

APD seems to be an important mediator of bloating and distension in IBS and FD, although other mechanisms also contribute. Certain patients, however, have isolated bloating and/or distension. APD has also been documented in pediatric patients, usually in association with aerophagia (14,15).These patients have functional bloating and distension as defined by the Rome IV criteria (Table 2) (16).

**Table 2.**
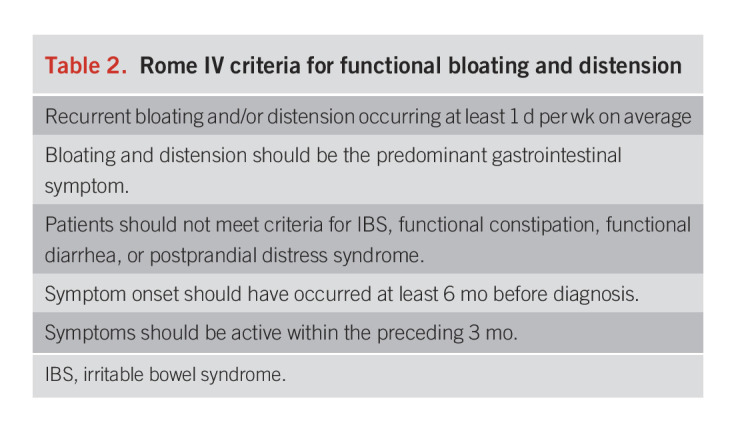
Rome IV criteria for functional bloating and distension

## EPIDEMIOLOGY

Because of the paucity of studies on APD, its prevalence is largely unknown. Presumably, given the high global prevalence of DGBIs (approximately 40%) and the association between DGBIs and APD, APD is likely a common and under-recognized phenomenon (17). APD may be more likely in women than in men, paralleling other DGBIs. Indeed, in the studies on APD to date, 93% of participants are women. As described above, APD is commonly associated with DGBIs, including IBS, FD, aerophagia (4,13,14), and functional bloating and distension, andis, thus, likely also associated with pathologies commonly comorbid in DGBIs, including anxiety, depression, trauma, and chronic pain syndromes (18).

## PATHOPHYSIOLOGY

Central to the pathophysiology of APD is the abnormal coordination of the diaphragm and abdominal wall musculature. EMG obtained during abdominal distension, either spontaneous episodes of distension or induced distension, consistently demonstrates increased activity of the diaphragm (causing diaphragmatic descent) coupled with decreased postural tone of the anterior abdominal wall, as compared with basal conditions (3,4,6,7). Under normal conditions, the activity of the diaphragm is counterbalanced by the costal wall to preserve pulmonary function (i.e., diaphragmatic descent is compensated by the descent of the costal wall) (19). By contrast, diaphragmatic descent during conditions of abdominal distension is associated with paradoxical elevation of the costal wall, driven by intercostal contraction, and resulting in hyperinflation of the chest. This condition functionally mimics status asthmaticus, explaining the shortness of breath characteristic of episodes of severe abdominal distension (6). Presumably, there is dysregulation along the gut-brain axis, which executes this abnormal response. Although the exact neurologic mechanism is not known, it has been hypothesized that APD develops as a maladaptive response to gastrointestinal pain or discomfort (13). Mechanosensors in the lumen of the gastrointestinal tract feed information about luminal contents and distension to the brain, which processes the information and in turn influences viscerosomatic physiology, such as by regulating neural and muscular activity and coordinating chest and abdominal wall musculature (20). DGBIs commonly feature central sensitization and visceral hypersensitivity, and so it is possible that APD arises as a reaction to pain derived from the gastrointestinal tract (21). Dysautonomia or abnormal autonomic responses have also been documented in DGBIs and associated with psychopathology and trauma and additionally supported by the fact that diaphragmatic breathing can improve symptoms (22).

## DIAGNOSIS

There are no current diagnostic criteria for APD (although as aforementioned, functional bloating and distension can be diagnosed by symptoms alone by the Rome IV criteria). The literature documenting APD has used various methods to detect its presence. Barba et al. (6) found that the morphological and volumetric differences observed in abdominothoracic CT scans obtained during periods of severe distension and periods with minimal or no distension are related to the functional differences in EMG activity in the abdominal walls. However, CT and EMG should be reserved for use in patients with suspected intestinal dysmotility and luminal pooling (6). Other studies have used abdominal inductance plethysmography to measure changes in abdominal girth, ultrasound to assess the movement of the diaphragm, and manometry to monitor esophageal and gastric pressures (1,13,19,23). Because the abnormal contraction of the diaphragm seems to be an important driver of APD, perhaps ultrasound could provide a simple, inexpensive, noninvasive, and reliable way to help identify paradoxical diaphragmatic movement reflective of APD (6,19).

## TREATMENT

At present, there are no standardized treatments of APD specifically; however, multiple treatments of functional abdominal bloating and distension have been studied and discussed elsewhere (6,24). In this study, we focus on treatments that specifically target the physiology that underlies APD.

### EMG biofeedback

Patients in the study by Barba et al. (6) used EMG activity as a visual signal for biofeedback. Using the real-time EMG signal, they were able to decrease EMG activity for the diaphragm and intercostal muscles, and this response was associated with the ascension of the diaphragm, reduction in abdominal girth, and improvement in the subjective sensation of abdominal distension (6). Similarly, in a subsequent placebo-controlled trial, Barba et al. (7) found that the biofeedback technique enabled patients with various DGBIs and symptoms of postprandial bloating to reduce intercostal muscle activity and increase anterior wall muscle activity, thereby reducing the sensation of abdominal distension and perceived girth. These data indicate that patients can be trained to control abdominophrenic postural tone, release the diaphragmatic blockade, and correct abdominal distension. Hence, biofeedback may be useful to correct APD and abdominal distension; however, the EMG technique used is complex and expensive, and there is currently extremely limited access and no standardized protocols.

### Diaphragmatic breathing

Diaphragmatic breathing is effective for treating aerophagia, belching, and rumination syndrome (25–28). This technique may also be effective for the management of bloating, because it targets the maladaptive somatic response. Patients are instructed to inhale slowly while protruding the abdomen, avoiding chest rise, and then exhale (29). Diaphragmatic breathing is indicated for 30 minutes after meals, placing one hand over the chest and the other over the abdomen (29,30). A major benefit of this treatment is that it is easy to learn and can be performed at home. Many gastroenterologists, physical therapists, and psychologists are also familiar with this technique, making it much more easily accessible for patients, but its value for the treatment of APD has not been established.

### Specific DGBIs treatments

Although other treatments of DGBIs have not been tested specifically for APD because shared mechanisms may contribute to each, treatments well established for DGBIs may be useful in the treatment of APD as well. Specifically, because central sensitivity and visceral hypersensitivity may play a role in APD pathogenesis, therapies that target these aspects of the pathophysiology could be of benefit. These include the low-fermentable oligosaccharides, disaccharides, monosaccharides and polyols diet (FODMAP) diet (which reduces intestinal luminal osmotic load, gas burden, and thereby distension), peripheral and psychological-based therapies such as cognitive behavioral therapy (CBT), and gut-directed hypnotherapy (31–35).

Central neuromodulators such as atypical antidepressants and gabapentinoids are useful for DGBIs symptoms. A recent placebo-controlled trial of 85 patients with IBS showed that treatment with pregabalin 225 mg twice daily significantly improved abdominal bloating (36). However, the role of neuromodulators in visible abdominal distension and APD remains to be established (32). CBT seems promising because it targets maladaptive behaviors and helps regulate psychological and physiologic responses to emotional and physical stimuli.

Finally, associated comorbidities and symptoms, including IBS, FD, pelvic floor dysfunction, endometriosis, anxiety, depression, and PTSD, should be appropriately treated. CBT may be particularly useful in patients with significant psychological comorbidities.

## CONCLUSION AND FUTURE DIRECTIONS

APD is characterized by the pathologic contraction and descent of the diaphragm and relaxation and protrusion of the abdominal wall. A series of studies indicate that this abnormal response contributes to distension in DGBIs, including IBS, FD, and functional bloating and distension. There are currently no diagnostic criteria for APD. Dynamic cross-sectional imaging can identify APD, but less expensive and easily accessible technologies, such as ultrasound, may be more useful in identifying and even treating APD. There are promising data to support the use of biofeedback to treat APD, but there are currently no standardized treatment protocols, and access to biofeedback is limited. Adjunctive treatments targeting central sensitization and visceral hypersensitivity may also be helpful. With the growing appreciation of APD and its contribution to DGBIs, future efforts should increase the understanding of APD, develop standardized diagnostic criteria, and propose evidence-based treatment recommendations.

## CONFLICTS OF INTEREST

**Guarantor of the article:** Fernando Azpiroz, MD, PhD.

**Specific author contributions:** J.D., S.T., and E.B.: study design, conduction of review, data analysis. F.A.: data interpretation.

**Financial support:** None to report.

**Potential competing interests:** None to report.
